# Temporal-Spatial Variation of Global GPS-Derived Total Electron Content, 1999–2013

**DOI:** 10.1371/journal.pone.0133378

**Published:** 2015-07-20

**Authors:** Jinyun Guo, Wang Li, Xin Liu, Qiaoli Kong, Chunmei Zhao, Bin Guo

**Affiliations:** 1 College of Geodesy and Geomatics, Shandong University of Science and Technology, Qingdao, 266590, China; 2 State Key Laboratory of Mining Disaster Prevention and Control Co-founded by Shandong Province and Ministry of Science & Technology, Shandong University of Science and Technology, Qingdao, 266590, China; 3 Chinese Academy of Surveying and Mapping, Beijing, 100830, China; ETH, SWITZERLAND

## Abstract

To investigate the temporal-spatial distribution and evolutions of global Total Electron Content (TEC), we estimate the global TEC data from 1999 to 2013 by processing the GPS data collected by the International Global Navigation Satellite System (GNSS) Service (IGS) stations, and robustly constructed the TEC time series at each of the global 5°×2.5° grids. We found that the spatial distribution of the global TEC has a pattern where the number of TECs diminishes gradually from a low-latitude region to high-latitude region, and anomalies appear in the equatorial crest and Greenland. Temporal variations show that the peak TEC appears in equinoctial months, and this corresponds to the semiannual variation of TEC. Furthermore, the winter anomaly is also observed in the equatorial area of the northern hemisphere and high latitudes of the southern hemisphere. Morlet wavelet analysis is used to determine periods of TEC variations and results indicate that the 1-day, 26.5-day, semi-annual and annual cycles are the major significant periods. The fitting results of a quadratic polynomial show that the effect of solar activity on TEC is stronger in low latitudes than in mid-high latitudes, and stronger in the southern hemisphere than in the northern hemisphere. But the effect in low latitudes in the northern hemisphere is stronger than that in low latitudes in the southern hemisphere. The effect of solar activity on TECs was analyzed with the cross wavelet analysis and the wavelet coherence transformation, and we found that there appears to be a strong coherence in the period of about 27 days. So the sunspot as one index of solar activity seriously affects the TEC variations with the sun’s rotation. We fit the TEC data with the least squares spectral analysis to study the periodic variations of TEC. The changing trend of TEC is generally -0.08 TECu per year from 1999 to 2013. So TECs decrease over most areas year by year, but TECs over the Arctic around Greenland maintained a rising trend during these 15 years.

## Introduction

The ionosphere is formed under the ionisation effect of extreme ultraviolet (EUV) radiation and solar X-ray, and is influenced by solar winds and geomagnetic activity. In addition, the lower atmospheres contribute to the variability of the ionosphere [1–2]. A variety of periodic and aperiodic variations could be observed in the ionosphere, which makes a serious impact on short wave communication, satellite communication and precise navigation with the Global Navigation Satellite System (GNSS), such as GPS [3]. GNSS as one contemporary technique to detect the ionosphere has many merits including the high spatial-temporal resolution (the spatial resolution is smaller than 1°×1°, and the temporal resolution is about 15 minutes), high station density (more than 400 IGS stations), good data quality and high precision [4–5]. With the rapid development of computerised ionospheric tomography (CIT), the total electronic content (TEC) of the large-scale ionosphere can be estimated precisely in the three-dimensional space [6]. So GPS technique is very useful for studying global TEC changes, and is the main technique for detecting TEC from the pseudo-codes and carrier phases [7–8]. In the 1980s, researchers started to estimate TECs based on the media dispersivity using GPS dual-frequency data.

TEC has complex temporal-spatial fluctuations including the periodic variation, such as the diurnal variation, monthly variation, and annual variation, and the momentary disturbance [9]. The global mean TEC from the Jet Propulsion Laboratory (JPL) (1998–2008) was calculated, and the mean data expressed strong solar cycle, annual/semiannual, and 27-day variations. The annual variations were found to be stronger in the southern hemisphere, and the semiannual phases and amplitudes were much stronger in the conjugate hemisphere [10–11]. The ionospheric seasonal characteristics in TEC during the declining activity phase of the 23 solar cycle in India were described systematically, and it indicated that seasonal variations in daytime TEC show a semiannual periodicity, and the spring equinox shows the highest TEC and the winter solstice the lowest [12]. Some seasonal anomalies have been described in detail [13–14], including winter or seasonal anomaly, semiannual anomaly, annual anomaly or December anomaly. In addition, other natural phenomena can cause a TEC momentary disturbance, such as a solar flare, magnetic storm, earthquake and so on [15–18]. The effect of solar activity on the ionosphere was investigated on a global scale, and it was found that the effect was stronger at day than at night, and was also more apparent in low latitudes than in high latitudes. In addition, the minimum effect appeared in lower latitudes around the dip equator, and the maximum effect appeared on both sides of the dip equator [11].

The latitudinal variations of TEC over Europe for the period from 1999 to 2001 indicated that TEC decreases monotonously towards the high latitudes, the latitudinal gradients are larger in winter than in summer [19], and the ionospheric climatological analysis revealed the mean TEC diminished from low latitudes to high latitudes in both hemispheres [13, 20]. The global distribution of vertical total electron content (VTEC) from GPS-based measurements represented that the global maximum TEC appears near the equator, and the diurnal variation of TEC peaks about 14:00 LT, decaying to a minimum just before sunrise. In addition, the equatorial anomaly phenomenon can be observed clearly in low-latitude regions, which usually appears between 10:00 and 13:00 LT [21]. TEC spatial distribution also contains regional characteristics. Such as the equatorial anomaly, which can be reasonably explained by the fountain effect [22–24]. The spatial structures of traveling ionospheric disturbances (TIDS) were observed in the mid-latitude ionosphere, and at night, most traveling structures in the mid-latitude ionosphere of the northern hemisphere tend to travel to the southwest [25]. TEC outside of the aurora region is higher than that in the aurora region, and TEC in the polar cap area is less than that in the aurora region [26]. However, the temporal-spatial evolutional law of ionosphere has not been systematically analyzed because of the differences in research purposes and data.

In this paper, we utilise the GPS-derived TEC data supplied by the Centre for Orbit Determination in Europe (CODE) from 1999 to 2013 to investigate the global spatial distribution and temporal variation of the ionosphere [27]. This analysis aims at elucidating some detailed features of the ionosphere, including the negative correlation between TEC and latitude, as well as annual, monthly and hourly variations. In addition, yearly variation of TEC will be investigated at the regional scale.

## Data and Methods

### TEC data sources

The international GNSS service (IGS) supplied TEC data with the time resolution of 2 hours determined from more than 350 IGS stations on a global scale. These TEC data can be used to study the spatial and temporal distribution of the ionosphere because of global data with the appropriate time resolution [28]. CODE as one of seven analysis centres of IGS has estimated TECs from the dual-frequency code and phase data since April 1998. In the current study, TEC data were derived from CODE (ftp://ftp.unibe.ch/aiub).

### Methodology

The observation equation of ionospheric delay is expressed as follows:

$$P_{i}=\rho +\frac{40.3}{f_{i}^{2}}\times F(\text{z})\times TEC+b^{s}-b^{r}+\Delta \;,\;\;\;\;i=1,2$$ (1)

$$\Phi_{i}\lambda_{i}=\rho -\frac{40.3}{f_{i}^{2}}\times F(\text{z})\times TEC-N_{i}\times \lambda_{i}+B^{s}-B^{r}+\Delta \;\;,\;\;\;i=1,2$$ (2)

where *i* is the number of signal frequency, *P* is the pseudorange, Φ is the carrier phase, *ρ* is the geometrical distance from the receiver to the satellite, *f* is the frequency, *F(z)* is the mapping function with respect to the elevation angle *z*, *b* ^*s*^ is the satellite circuit delay error of pseudo-range, *b* ^*r*^ is the receiver circuit delay error of pseudo-range, *B* ^*s*^ is the satellite circuit delay error of phase, *B* ^*r*^ is the receiver circuit delay error of phase,*λ* is the wave length, *N* is the integer ambiguity, and Δis the correction term including the receiver clock error, satellite clock error, troposphere delay, multipath correction and so on.

TEC can be calculated according to the pseudorange and phase data in the following way:

$$TEC=-\frac{1}{40.3F(\text{z})}\frac{f_{1}^{2}f_{2}^{2}}{f_{1}^{2}-f_{2}^{2}}(P_{4}-dcb)$$ (3)

$$TEC=\frac{1}{40.3F(\text{z})}\frac{f_{1}^{2}f_{2}^{2}}{f_{1}^{2}-f_{2}^{2}}(L_{4}-\Delta Amb-DCB)$$ (4)

Where *P*_4_ = *P*_1_ - *P*_2_, *L*_4_ = Φ_1_ *λ*_1_ - Φ_2_ *λ*_2_, $dcb=(b_{1}^{s}-b_{2}^{s})-(b_{1}^{r}-b_{2}^{r})$ is the receiver hardware delay, $DCB=(B_{1}^{s}-B_{2}^{s})-(B_{1}^{r}-B_{2}^{r})$ the satellite hardware delay, and Δ*Amb = N*_2_ *λ*_2_ - *N*_1_ *λ*_1_.

The global ionosphere map (GIM) with the time resolution of 2 hours and the spatial resolution of 5°×2.5° can be provided by CODE. TECs of global grids are reconstructed by fitting the vertical TECs of IGS stations with the spherical harmonics function [29]

$$TEC\left(\alpha ,\beta \right)\text{=}\sum_{n=0}^{n_{\text{max}}}\sum_{m=0}^{n}P_{nm}(\alpha )\left[a_{nm}\text{cos}(m\beta )+b_{nm}\text{sin}(m\beta )\right]$$ (5)

where *α* and *β* are the latitude and longitude respectively, *n*_max_ is the highest degree, *P*_*nm*_ (*α*) is the normalised associated Legendre function, *a*_*nm*_ and *b*_*nm*_ are the spherical harmonics coefficients, and *n* and *m* are degree and order, respectively.

To transform the slant TEC into the vertical TEC, the mapping function of the single layer model was used before 9 September 2001 [30]:

$$F(z)=\frac{1}{\text{cos}\;z^{\prime }}$$ (6)

Where sin *z' = R* sin *z* / (*R* + *H*), *R* = 6371 km, and *H* = 450 km. Since then, Mannucci et al. [22] modified the single layer model and selected sin *z' = R* sin(*kz*) / (*R* + *H*) in which *H* = 506.7 km, and *k* = 0.9782.

Now, more than 350 GPS stations collect data to estimate GIM [31]. Each GIM in the solar-geomagnetic reference frame is transformed into the Earth-fixed reference frame with a spatial resolution of 5° in longitude and 2.5° in latitude, and a temporal resolution of 2 hours. TEC in a solar-geomagnetic reference frame is modeled with bicubic splines on a spherical grid. Kalman filtering is used to solve simultaneously TEC and instrumental biases. The detailed TEC deriving procedure is described by Mannucci et al. [22].

## Results and Discussion

### Spatial distribution of TEC

In the study, we computed the sum of the TEC from 1999 to 2013 on each grid, and then the averaged TEC on each grid can be calculated by the arithmetical average method. We utilised the averaged TEC on each grid to analyse the spatial distribution of global TEC with the resolution of 5°×2.5°.

**Fig 1:**
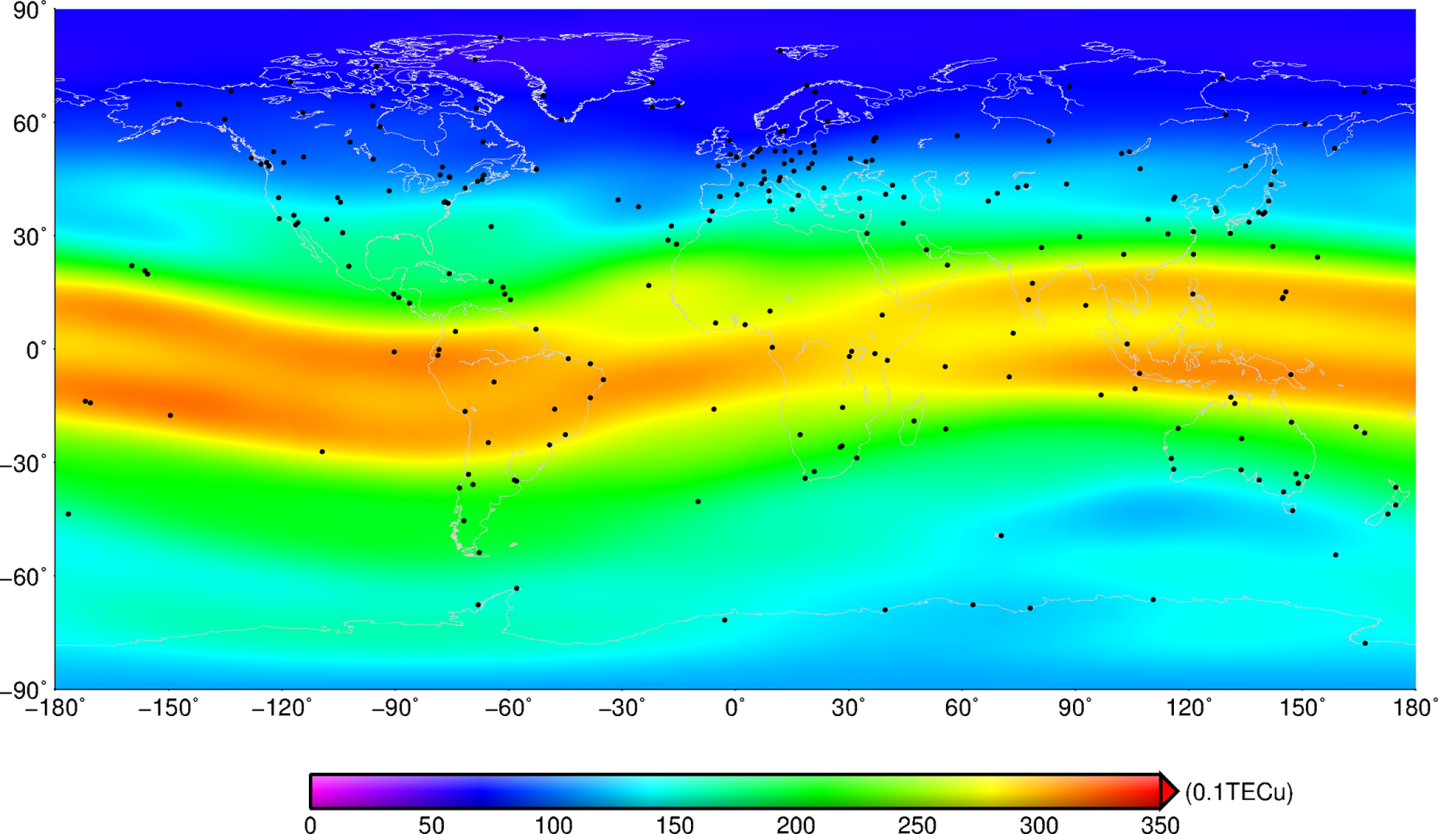
Spatial distribution of mean global TECs from 1999 to 2013. Black points denote the stations that are used to estimate the GIM.

**Fig 2:**
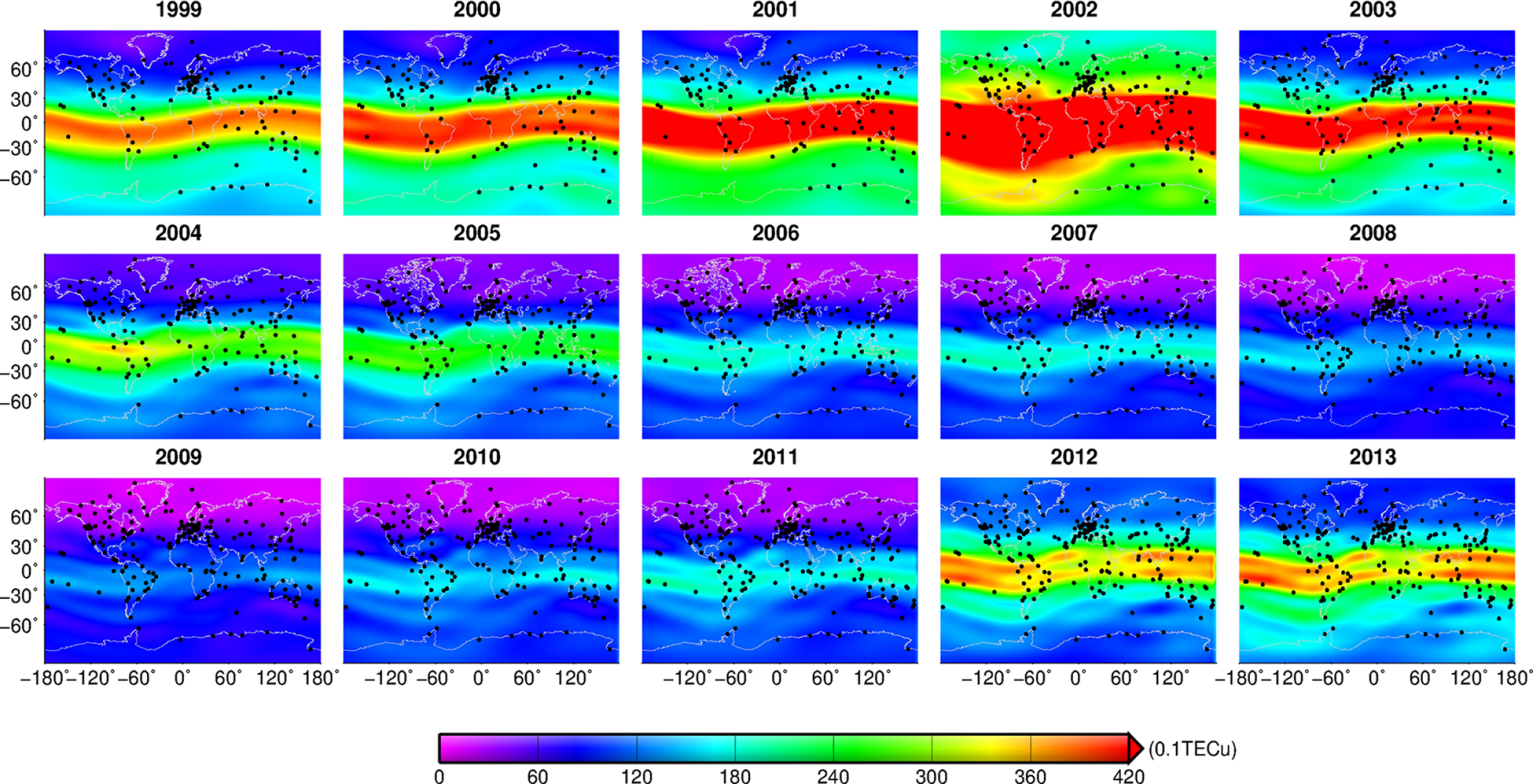
Annual TECs from 1999–2013. Black points denote the stations that are used to estimate the GIM.

As shown in Fig 1, there is obviously a regional characteristic in the global spatial distribution of the ionosphere. The amount of TEC in the low-latitude region is maximum, and the amount of TEC in the high latitudes is minimum. The amount of TEC near the equator is up to more than 30 TECu, which is three times greater than that at the high latitudes. On the whole, TEC should decrease progressively from the low latitudes to the high latitudes with some abnormal phenomena on local areas. For instance, the minimum value of averaged TEC appears in Greenland and its surrounding area. TEC has a slightly different distribution for the same latitudes. TEC over the equatorial zone has a slightly irregular distribution. For example, there is a remarkable double-peak structure in the equatorial area, and in the longitude ranges around longitude 0° the double peaks are sharpness, which is in accordance with other research results [14]. In addition, the double peak structure in the region between -60°and 60°of longitude is fuzzy, and the electron concentration in the region is lower than that in other regions of equatorial zone. The positions of IGS stations used to compute the GIM were marked with black points in Fig 1, and more than 10 stations were distributed in this region. Compared with the number of IGS stations in other areas of equatorial ionization anomaly region, such as the Pacific Ocean, the station density in the region was not lower. As shown in Fig 2, there were an increasing number of IGS stations were used to compute the GIM from 1999 to 2013. In 1999–2003, the number of stations in the region between -60°and 60°of longitude was relative lower, and the hardwares were not perfect. In the period, the distinct double peak structure can be observed in the particular region. With the development of GNSS technique and hardware level, more stations were used to estimate the global TEC, and the double peak in the particular region gradually disappear. Especially after 2008, the phenomenon was more obvious. The analysis results indicated that the phenomenon in the particular region was a real effect, not caused by inadequate number of available stations. Furthermore, TECs over the mid-high latitudes of northern hemisphere are obviously lower than that over the same latitude regions of southern hemisphere. In the middle and high latitude areas of northern hemisphere, the mean value of TEC is less than 15 TECu and the TEC over the high latitudes is only about 8 TECu. But there is a significant difference between the mean TECs in the middle and high latitude areas of the southern hemisphere. TEC here is about 15 to 25 TECu, and TEC in the high latitudes is two times greater than that in the same latitude of northern hemisphere.

The difference of TEC over the northern and southern hemispheres is mainly distributed in polar regions. In polar regions, TEC generation mechanism is different from that in the mid-low latitude zones, and it is mainly caused by the ionisation effect of solar radiation, magnetosphere electric fields, electron precipitation over the polar region, aurora particle precipitation, polar plasma convection and density change of neutral atmospheric composition [32]. Among them, the ionisation effect of solar radiation, magnetosphere electric fields and aurora particle precipitation are the dominant factors. Firstly, solar radiation is the primary source for producing electrons; secondly, the neutral atmosphere is ionised by aurora particle precipitation from magnetosphere, and the electron concentration is changed [33]. Furthermore, magnetosphere electric fields spread to the polar ionosphere, which lead to the horizontal transportation of plasma and the redistribution of electron concentration [34–35]. As a consequence, the above factors contribute to the different distribution of TEC over the northern hemisphere and southern hemisphere in high latitude regions.

### Temporal variation of TEC

Understanding the temporal variations of TEC will play an important role in creating the global ionospheric model. We used the global TEC data from 1999 to 2013 determined with GPS data to build TEC time series on different time scales, and then analyze the significant cycles using the wavelet analysis.

#### TEC inter annual variation

We used the global TECs from 1999 to 2013 to extract the annual mean TEC of each grid with the resolution of 5°×2.5°, and the stations used to compute the global GIM were marked with black points, as shown in Fig 2. TECs reduce gradually from the low-latitude region to high-latitude region each year in the spatial distribution. TECs began to increase in 1999 and reached a maximum in 2002 in the temporal distribution. The TEC variation over the equatorial region is most significant, and the mean TECs at mid-high latitudes in the southern hemisphere are greater than that in the northern hemisphere. Global TECs began to diminish from 2003 to 2009, and TECs in 2009 were up to the trench whose mean value was less than 12 TECu. With the increase of stations used to estimate the GIM, more detail characteristics can be observed. For example, the double peak structure in the area between -60°and 60°of longitude becomes fuzzy since 2004, especially in 2012 and 2013. After 2009, TECs began to increase all over world, so the time span between the crest and the trench is about 7 years.

#### TEC inter-monthly variation

**Fig 3:**
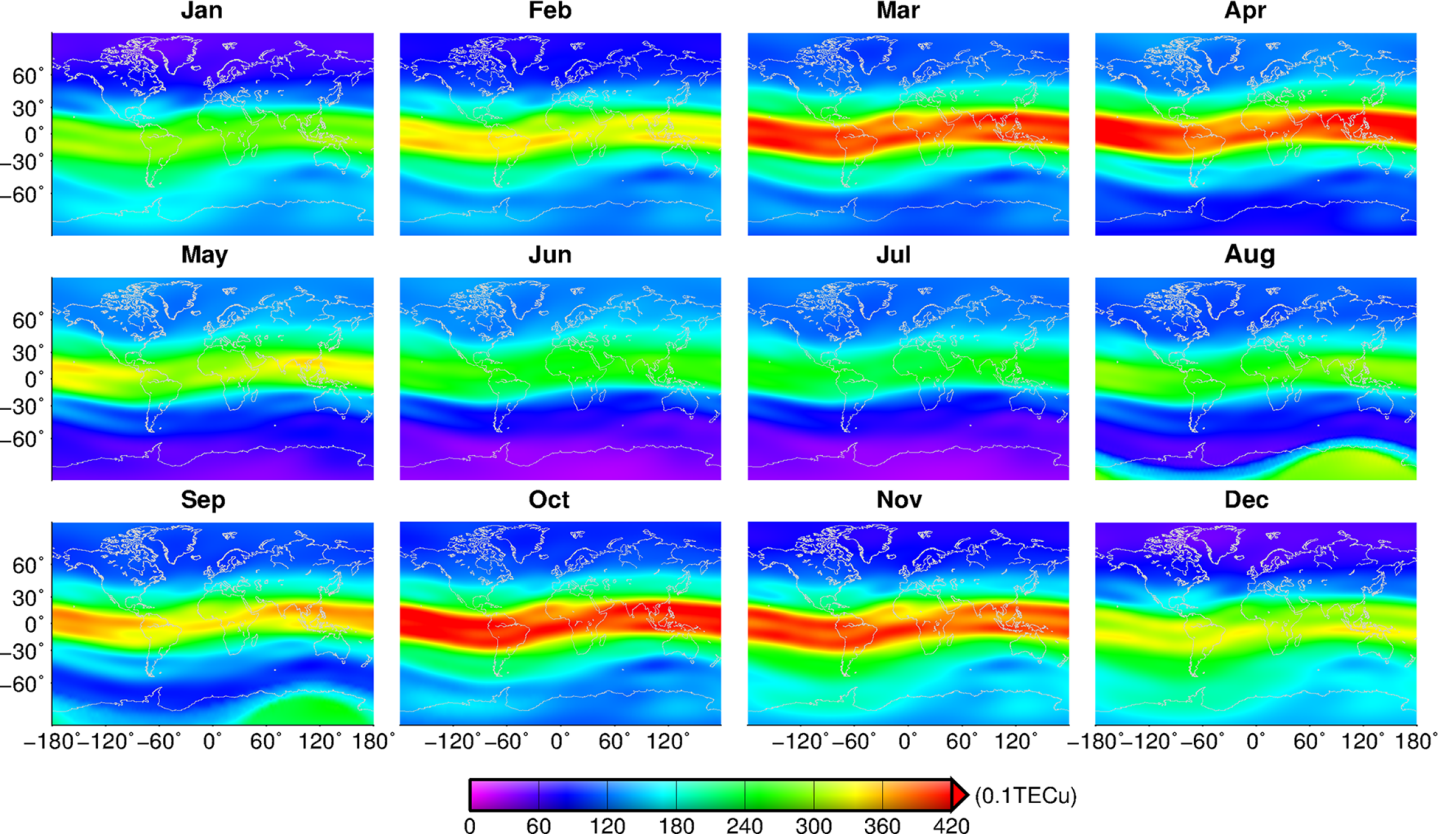
Monthly TECs from January to December.

We use the global TECs from 1999 to 2013 to extract the monthly mean TEC of each grid with the resolution of 5°×2.5°, as shown in Fig 3. There may be a periodical characteristic in the inter-monthly variation of global TECs. TECs in March, April, October and November are significantly greater than those in other months, which is consistent with other research that shows that the peak TEC appears in equinoctial months in most regions [36], and it also corresponds to the semiannual variation of TEC [14]. The semiannual variation of TEC is more obvious in the equatorial region and has the same characteristics as the geomagnetic equator. This may be caused by the interaction of solar zenith distance, atmospheric circulation over the high-latitude zone, and the changing proportion of oxygen and nitrogen *[O/N*_*2*_ *]* [15, 37–38]. The averaged TECs between the two solstices are not symmetrical along with the magnetic equator, being generally greater in the southern hemisphere than in northern hemisphere. Furthermore, the TECs in the northern equatorial regions in summer are lower than those in winter, which is in agreement with the analysis of topside plasma density from 1996 to 2005 [39]. TECs over the Antarctic in August and September are obviously greater than those in January and February, which is known as the winter anomaly in the polar zone. However, in previous research, the winter anomaly only appears in near-pole regions in the northern hemisphere [36]. The winter anomaly at high latitudes in the southern hemisphere is rarely found. The global amplitude of *[O/N*_*2*_ *]* annual variation estimated with the MSIS90 model has symmetrical distribution with the geomagnetic latitude, and the phase analysis indicates that the maximum of *[O/N*_*2*_ *]* occurs in the local winter over the high-latitude regions, which is consistent with the polar winter anomaly of TEC [40]. This suggests that the variation of *[O/N*_*2*_ *]* in the atmosphere is one important cause of the winter anomaly in the polar region [36].

#### TEC inter-hourly variation

**Fig 4:**
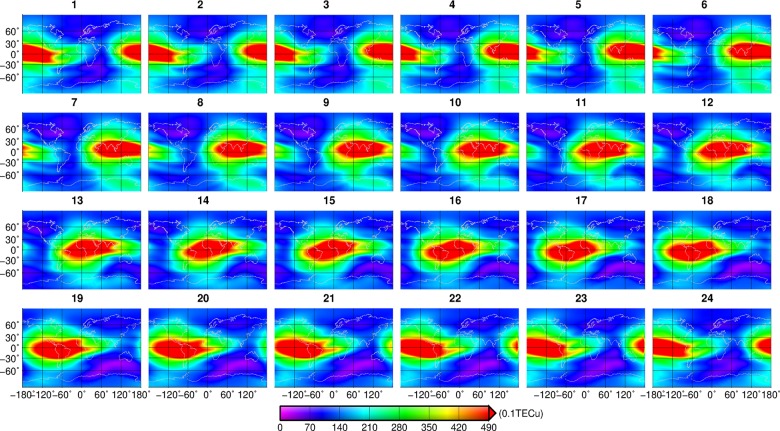
Hourly TECs in UTC.

CODE (ftp://ftp.unibe.ch/aiub) released GIM at odd hours before 3 November 2002, and after that, even hours instead of odd hours. Therefore, the CODE GIM from 2003 to 2013 are utilized to compute the mean GIM at even hours, then the mean GIM at every hour could be calculated with the resolution of 5° (in longitude)×2.5° (in latitude) by the linear interpolation, as shown in Fig 4. The red area in Fig 4 standing for the high TEC concentration starts to move to the north from 1 hour UTC and reaches the northernmost at 6 hours UTC. The high TEC area moves to the south and reaches the southernmost at 19 hours UTC. Then the red area starts again to move to the north. The cycle results in the high electronic concentration zone and the equatorial anomaly zone. The high concentration zone does not distribute regularly along the equator, and two crests appear in Southeast Asia and the North Africa, respectively. The northern crest is located at (120°E, 23.5°N), and the southern crest is located at (80°W, 32.5°S) where the latitudes are asymmetric, as shown in Fig 1. The generator field of the ionospheric E layer over the middle- and low-latitude regions can be mapped to the F_2_ layer along the magnetic line of force in the daytime, which drives the plasma of the F_2_ layer to drift along the direction perpendicular to the magnetic line of force. The magnetic lines of force over the geomagnetic equator region horizontally step across the geomagnetic equator and connect the northern and southern hemispheres, which makes the plasma move upward and then spread to 15°-latitude sectors on both sides of the magnetic equator under the effect of gravity to the two-crest shape [41].

#### Temporal variation TEC in local regions

**Fig 5:**
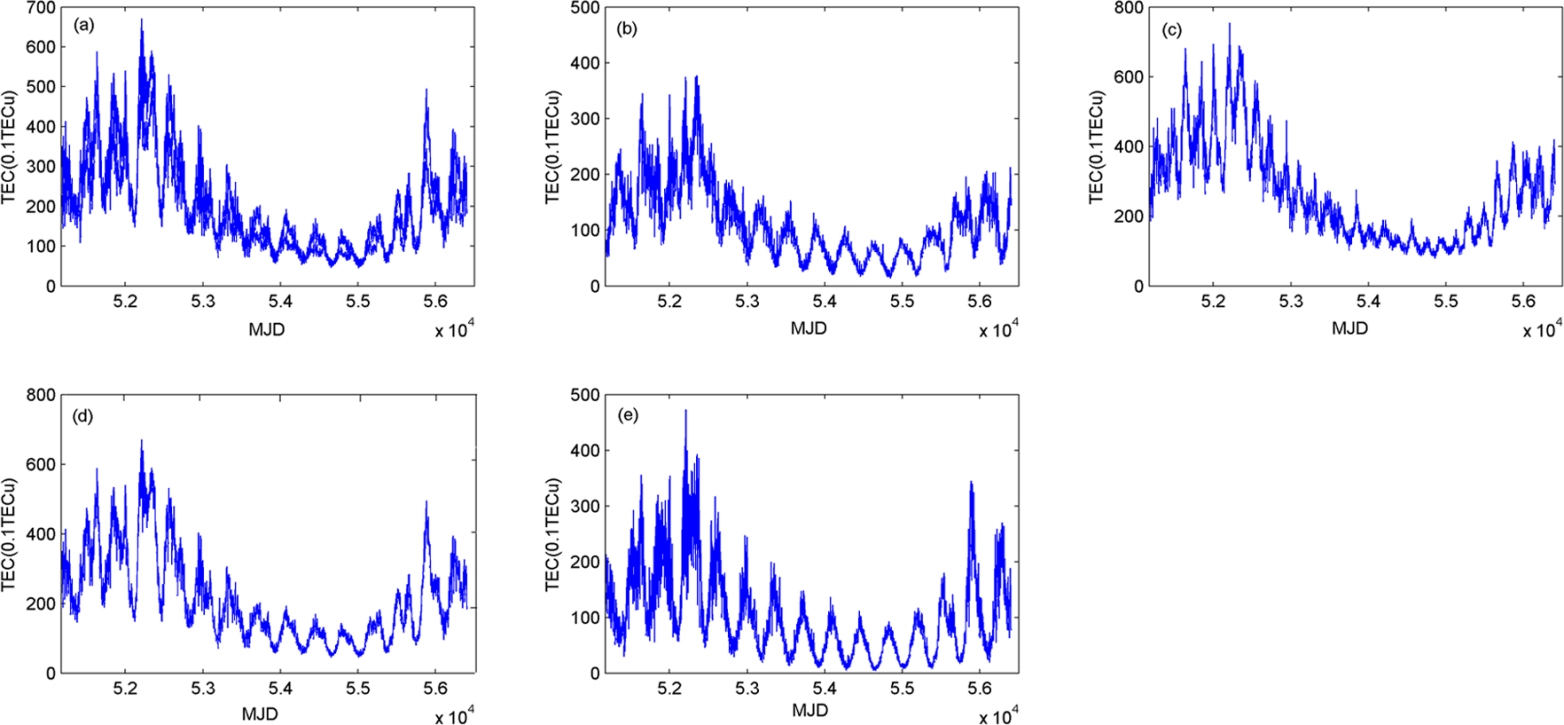
Time series of TECs in the global and local regions. (a) is the time series of global TEC. (b) is TEC time series on the northern region over 45°N. (c) is TEC time series over the local region between 45°N and the equator. (d) is TEC time series over the local region between the equator and 50°S. (e) is TEC time series over the southern region over 50°S.

Based on the spatial distribution of TEC and the electron density, the global ionosphere is divided into four regions, that is, the northern region of over 45°N, the region between 45°N and the equator, the region between the equator and 50°S, and the southern region of over 50°S. We use the global TECs from 1999 to 2013 with the time resolution of 2 hours to extract the daily mean TECs of each region and the global, as shown in Fig 5.

The TEC variations over these four regions and the global have the same trend but the different amplitudes. The TEC crest appeared in 52400 MJD (2002), the trough was present in 54900 MJD (2009), and another crest again appeared in 55900 MJD (2013). Comparing the time series of four regions, we can see that TECs over the low-latitude zone are greater than those over the high-latitude zone, as shown in Fig 5. TECs over the mid- and low-latitude zones in the northern hemisphere are greater than those over the same zones in the southern hemisphere, as shown in Fig 5c and 5d. TECs over the high-latitude zone in the southern hemisphere are greater than those over the same zone in the northern hemisphere, as shown in Fig 5b and 5e. This is consistent with the spatial distribution of global TECs in Fig 1.

### Wavelet analysis on TEC time series

**Fig 6:**
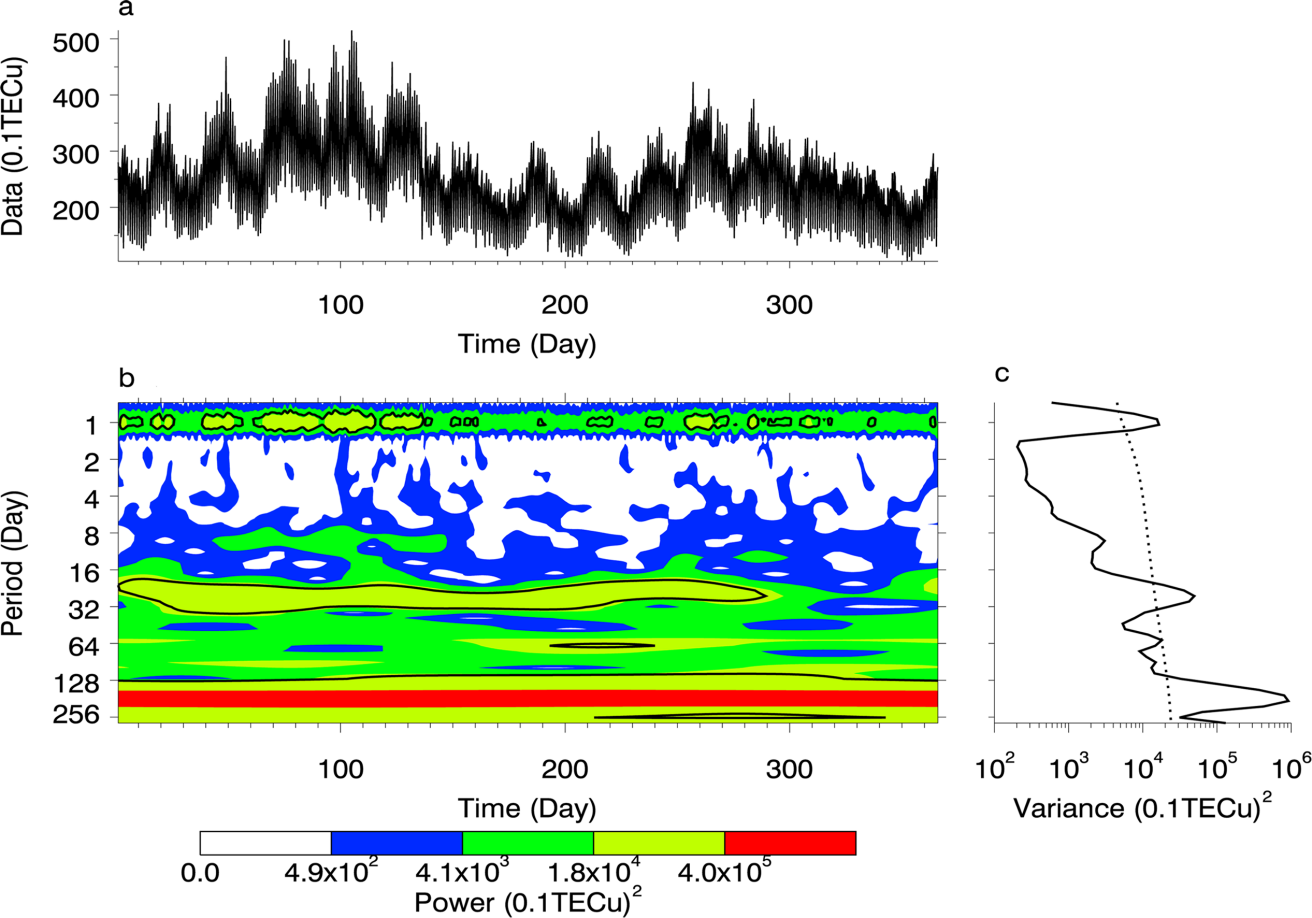
Periodic spectrum of TEC set A. (a) is the time series of TEC. (b) is the power spectrum with Morlet wavelet. (c) is the power with the confidence of 95%.

**Fig 7:**
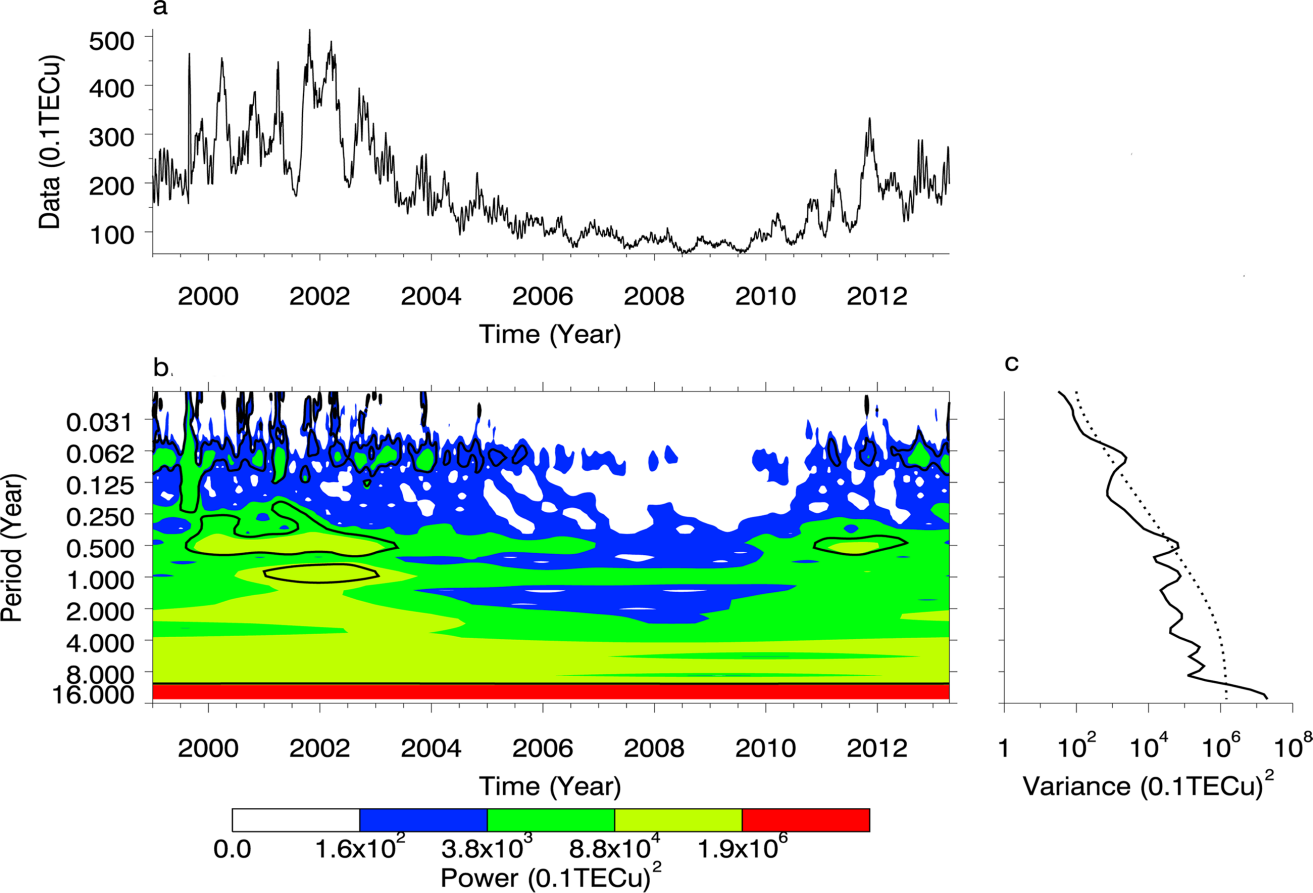
Periodic spectrum of TEC set B. (a) is the time series of TEC. (b) is the power spectrum with Morlet wavelet. (c) is the power with the confidence of 95%.

In order to detect the long period and the short period based on the ionosphere characteristics, we constructed two time series of TECs, that is, data A including the global TECs with the time resolution of 8 hours estimated by CODE in 2005, and data B including the global TECs with the time resolution of 3 days from 1 January 1999 to 15 April 2013. We used the Morlet wavelet to analyse data sets A and B, as shown in Figs 6 and 7.

In Fig 6 we can see there are three obvious periods, such as 1 day, 26.5 days and 187.7 days, and the spectral values of 1-day, 26.5-day, and 187.7-day variations all pass the confidence line of 95%. From Fig 7 we can see that there are periods of 0.007 year, 0.5 year, 1 year, 2.29 years, 5.13 years, 8 years, and 11 years, among which the spectral values of 0.007-year, 0.5-year and 11-year variation pass the confidence line of 95%. Therefore, we can determine that periods of 1 day, 26.5 day (0.007 year), 0.5 year and 11 years are significant over the total time span and the 1-year period is only significant over the local time span.

### Periodic analysis of TEC time series with the least squares spectral analysis

**Fig 8:**
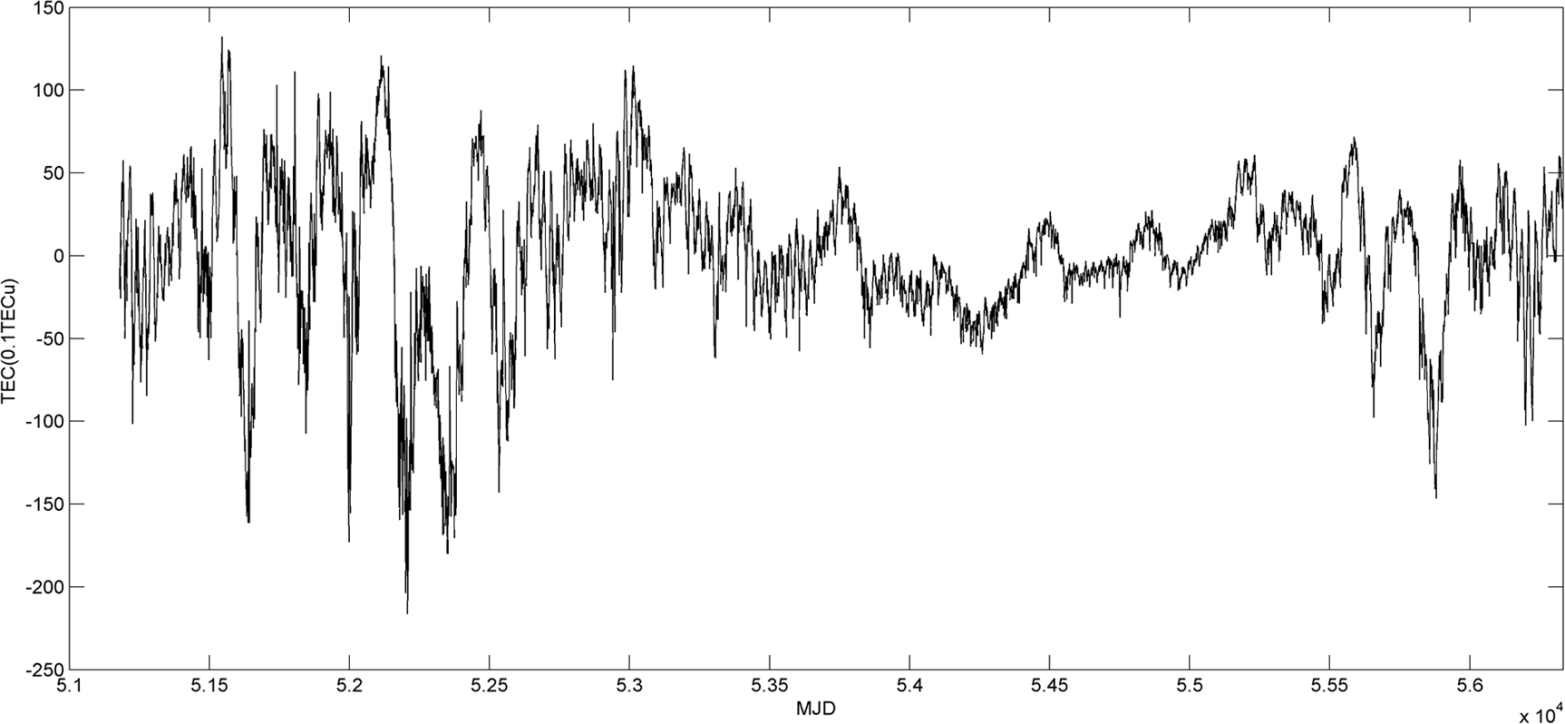
Residual TEC sequence.

There are five significant cycles of TEC variations, that is, 1 day, 26.5 days, semiannual, annual, and 11 years by the wavelet analysis. The least squares spectral analysis is used to estimate the trends, amplitudes, and phases of TEC time series in each grid by fitting the polynomial equations with unknown periodic parameters [42], that is,

$$y=\sum_{j=0}^{m}a_{j}t^{j}+\sum_{i=1}^{n}b_{i}\text{sin}(\omega_{i}t+\varphi_{i})$$ (7)

where the first term on the right hand is the aperiodic changes in which *m* is the maximum power, and here *m* = 2, and the second term is the summation of cyclical variations in which *n* is the maximum number of cycles, and here *n* = 5. *a*_*0*_ is the bias of time series, *a*_*1*_ is the linear trend, *a*_*2*_ is the change rate, *b*_*i*_ is the amplitude, *ω*_*i*_ is the frequency, and *φ*_*i*_ is the initial phase. The least squares method is used to estimate the unknown parameters in Eq 7 to fit the time series of TEC. The residuals between the fitting values and the real TECs are shown in Fig 8, and the statistical results are listed in S1 Table.

As shown in Fig 8, most of absolute residuals are below 10 TECu. The absolute residuals in 2000–2002 and 2010–2013 are larger, which are caused by the severe solar activity. The absolute residuals are decreased with the weakening solar activity in 2005–2009.

**Fig 9:**
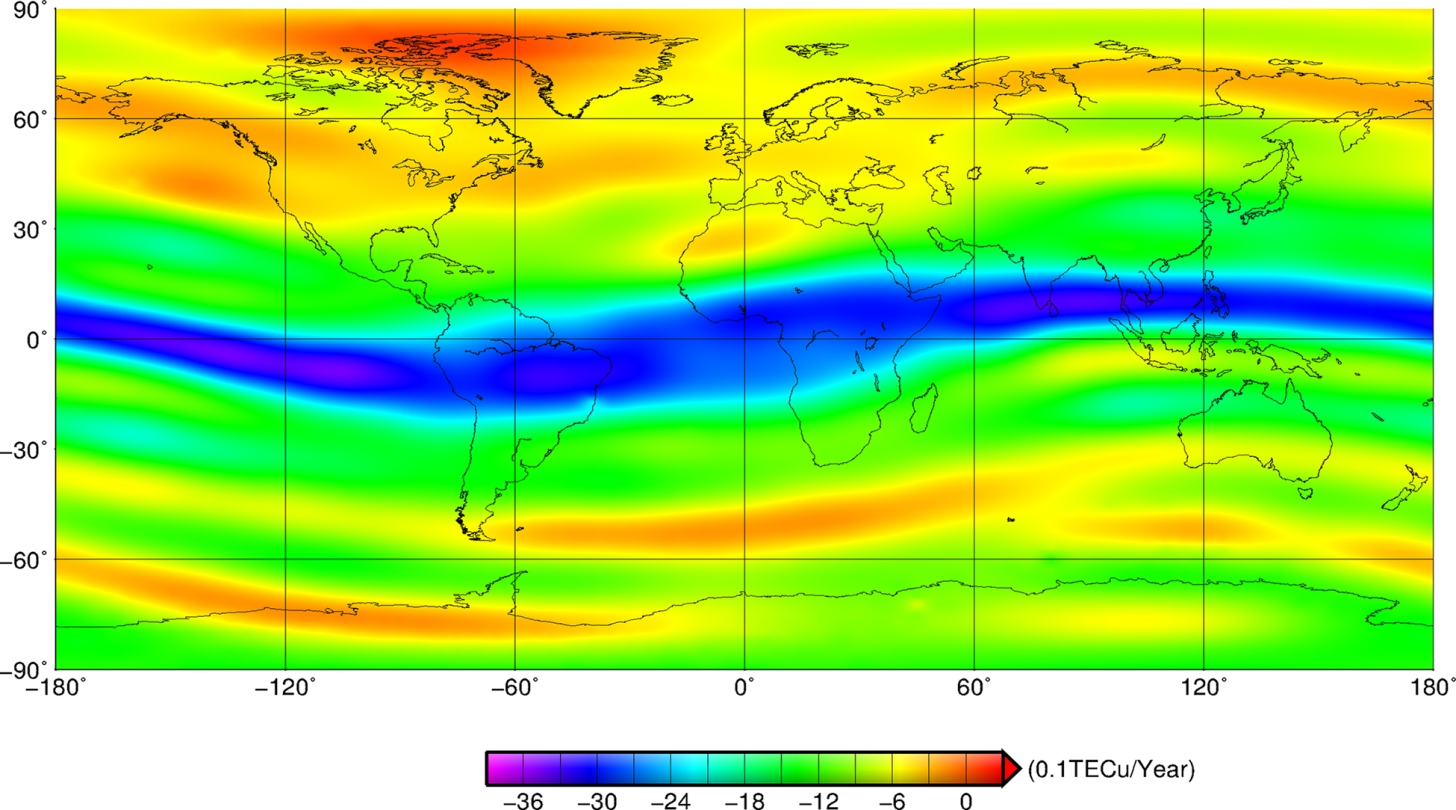
Trend of global TEC from 1999 to 2013.

The trend is the focused parameter in our study, which reflects the linear change of TEC from 2009 to 2013. It is significant for the TEC prediction and analysis of global climate change. The trends at all grids can be estimated with the least squares spectral analysis, as shown in Fig 9.

Also, as shown in Fig 9, the TECs over most regions present the trend of decreasing year by year. The TEC reductions in equatorial regions are maximum, with -2.4 to -3.9 TECu per year. The TEC trend in the equatorial regions is consistent with the inter-annual variation and the inter-monthly variation, which is obviously related to the geomagnetic equator. The reductions decrease gradually from the equator to the high latitude regions, which relates to the intensity of solar activity. The solar activity is very severe up to the maxima in 2002, and to the second maxima in 2013. So the amount of ultraviolet ray and X-ray is smaller in 2013 than in 2002, which is the dominating factor that the TECs of most regions generally present the decreasing trend.

The intensive solar activity has the most important effect on the middle and low latitude regions for creating a large number of electrons. In the period of subdued solar activity, the maximal reduction also emerges in the middle and low latitude regions. The tropic is about 23°26´ so that TECs are lower in the mid- and high-latitude regions because of shorter sunlight time. This is the reason for which the trend diminishes from the low latitude region to the high-latitude region. Though the global TEC presents the diminishing trend during these 15 years, the anomalies also exist in local regions. For example, TECs over Greenland increase about +0.3 TECu per year.

### Effect of solar activity on TEC

The atmosphere absorbs the solar ultraviolet and X-ray to form the ionosphere by the radiation ionising in the space environment around the Earth. The impacts of solar activity on the ionosphere always attract widespread attention [43]. Liu [21] described the characteristics of influence of solar activity on the ionosphere in different aspects, including features around the F2 peak, and features at different altitudes. Moussas et al. [44] reviewed the periodic solar activity whose significant cycles include 22 years, 11 years (Schwabe cycle), 5 years, 2 years, 1.7 years, and 1.3 years, in addition to the cycle of 27 days. Data of the sunspot number (SSN) as one important index of solar activity provided by the Solar Influences Data Analysis Centre (SIDC), Solar Physics Research Department (http://sidc.oma.be/sunspot-data/dailyssn.php) are used to analyze the effect of solar activity on TECs.

#### Correlation between SSN and TEC

To investigate the effect of solar activity on the ionosphere over different latitude ranges, we evaluated the regional daily mean TEC in both hemispheres at low latitude (0°-30°), middle latitude (30°-60°), and high latitude (60°-87.5°). We indicate ${\bar{TEC}}_{h}^{N}$ for the regional mean TEC averaged at high latitudes (indicated by the subscript *h*) in the northern hemisphere (indicated by the superscript *N*), and other variables will be listed in Table 1.

**Table 1:** Variables used in this article and their meaning.

Variable	Meaning
${\bar{TEC}}_{l}^{N}$	TEC over northern low latitudes (0°-30°N)
${\bar{TEC}}_{l}^{S}$	TEC over southern low latitudes(0°-30°S)
${\bar{TEC}}_{m}^{N}$	TEC over northern middle latitudes(30°-60°N)
${\bar{TEC}}_{m}^{S}$	TEC over southern middle latitudes(30°-60°S)
${\bar{TEC}}_{h}^{N}$	TEC over northern high latitudes(60°-87.5°N)
${\bar{TEC}}_{h}^{S}$	TEC over southern high latitudes(60°-87.5°S)

**Fig 10:**
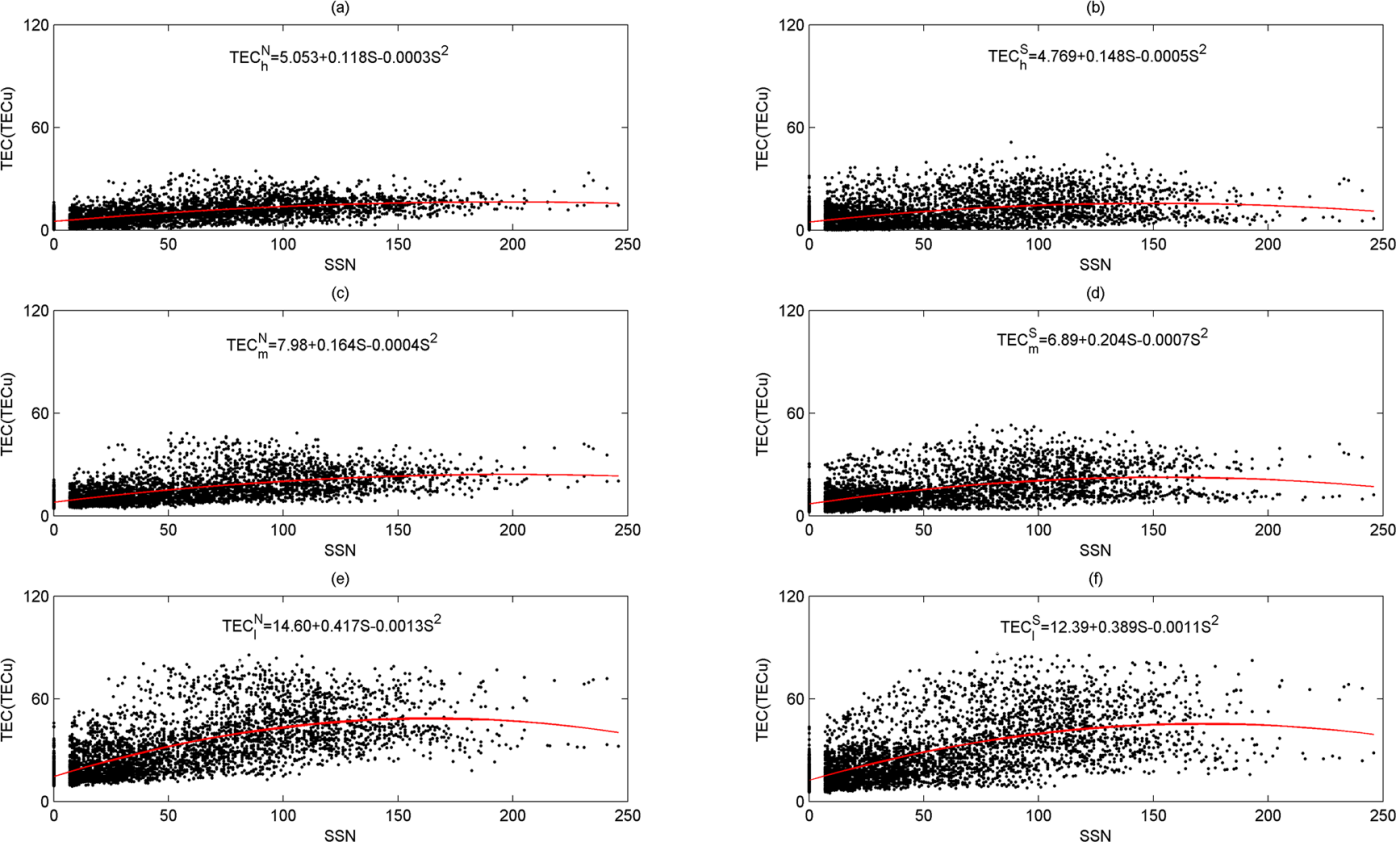
Dependence of the daily mean TEC on SSN in 1999–2013. the daily averages of mean TEC are averaged in both hemispheres at low latitudes (0°-30°), middle latitudes(30°-60°) and high latitudes(60°-87.5°). The superscripts *N*, *S* denote the northern hemisphere, southern hemisphere, respectively, the subscripts *l*, *m*, and *h* denote low, middle, and high latitudes. The variable S denotes sunspot number. The red curves denote the fitting results.

The daily mean TEC over different latitudes in both hemispheres from 1999 to 2013 are plotted as a function of the SSN in Fig 10. The second-order polynomial is applied to fit the function to show the effect of solar activity on the daily mean TEC averaged on three latitude bands. The red curve denotes the fitting results in Fig 10. We will focus on the coefficient of the first-order polynomial that represents the strength of the effect between SSN and TEC. As shown in Fig 10, the coefficients of the first-order polynomial at three latitude bands have a negative relation with latitude. For example, the coefficients at low latitudes, middle latitudes, and high latitudes in the northern hemisphere are 0.417, 0.164, and 0.118, respectively. The coefficient at low latitude is two to three times larger than that at mid-high latitudes. The variation tendency is also observed in the southern hemisphere, which indicates TECs over low latitudes are more sensitive to solar activity than those over mid-high latitudes. Furthermore, the mean TEC sensitivity to solar activity at the same latitude in both hemispheres is somewhat different. The coefficients at middle latitudes in the northern hemisphere and the southern hemisphere are 0.164 and 0.204, respectively, and at high latitudes, they are 0.118 and 0.148. It is clear that TECs in the southern hemisphere are more sensitive to solar activity than those in the northern hemisphere at mid-high latitudes. However, the change rule at low latitudes is unique, and the coefficient in the northern and southern hemispheres are 0.417, 0.389, respectively, which shows that the solar activity sensitivity of TEC is stronger in the northern hemisphere than in the southern hemisphere.

#### Periodic analysis of sunspots

**Fig 11:**
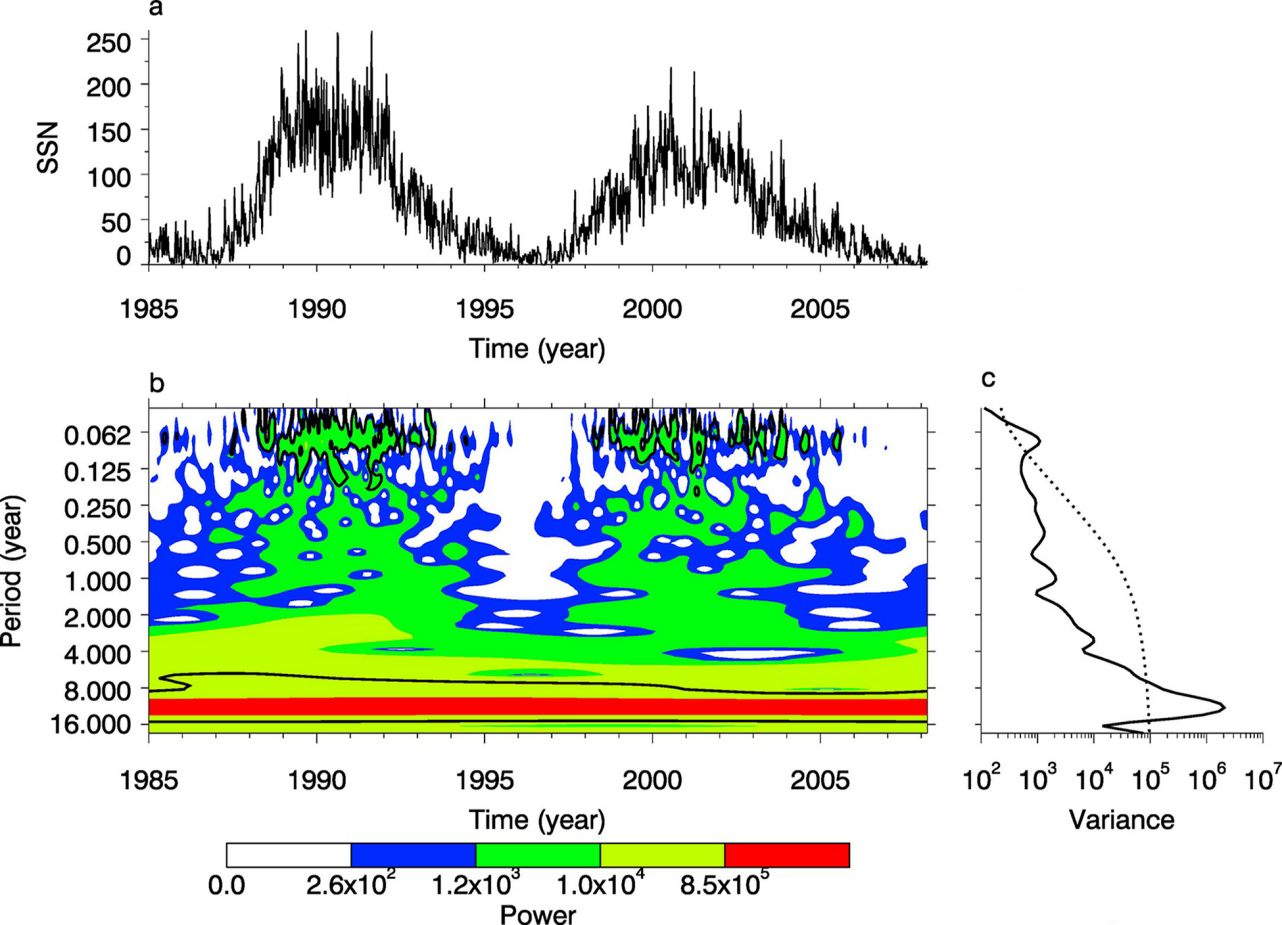
Periodic spectrum of SSN set A. (a) is the time series of SSN. (b) is the power spectrum with Morlet wavelet. (c) is the power with the confidence of 95%.

**Fig 12:**
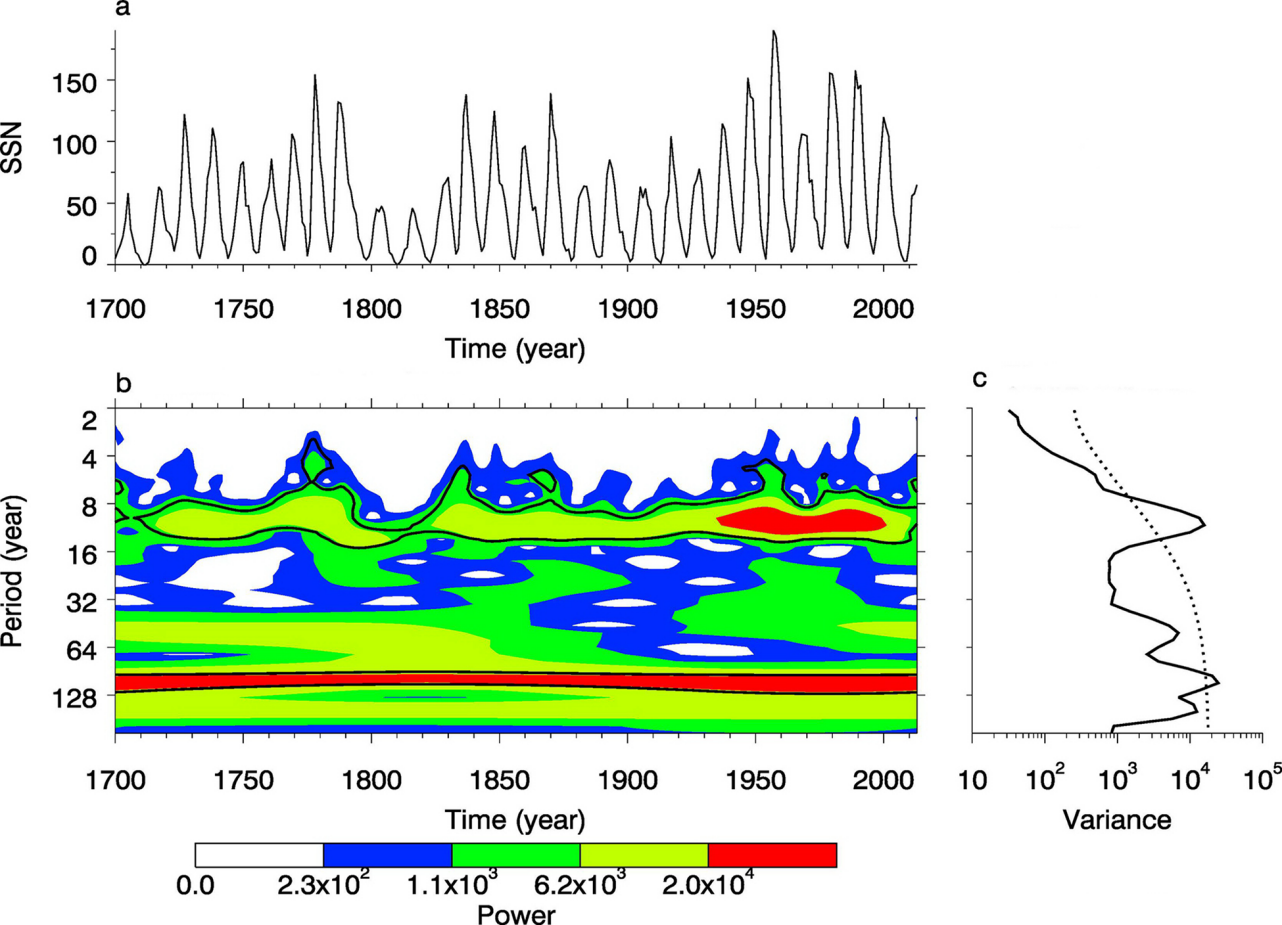
Periodic spectrum of SSN set B. (a) is the time series of SSN. (b) is the power spectrum with Morlet wavelet. (c) is the power with the confidence of 95%.

We use both datasets of SSN to systematically analyze the periods of sunspots to further study TEC variations, that is, dataset A including the daily mean values of SSN from 1985 to 2009, and set B including the annual mean values of SSN from 1700 to 2009. The Morlet wavelet was used to analyze these two sets, as shown in Figs 11 and 12.

As shown in Fig 11b, the periodic spectrum of sunspot number is significant in the periods of 0.062–0.125 years, 1–1.5 years, 2–4 years and 8–16 years. From Fig 11c, we can find there are four cycles, that is, 0.075 years (27.4 days), 1 year, 3.4 years and 11.04 years, and the spectral powers of 0.075-year and 11.04-year pass the 95% confidence line. As shown in Fig 12, the periodic spectrum of sunspot number is significant in these periods: 8–16 years, 32–64 years, and 64–128 years. From Fig 10c, we can find there are mainly three cycles, that is, 11.04 years, 52.7 years and 111.6 years.

#### Relationship between sunspot and TEC with the cross wavelet analysis

In order to further analyze the effect of SSN variation on TEC, we use the cross wavelet and wavelet coherence transformation to get the resonant periods between them with daily SSNs and TECs from MJD 51179 to MJD 56403 (1 January 1999 to 21 April 2013); the SSN data were provided by SIDC with the time resolution of 1 day, and the TECs were daily mean values of CODE-GIM from 1999 to 2013.

**Fig 13:**
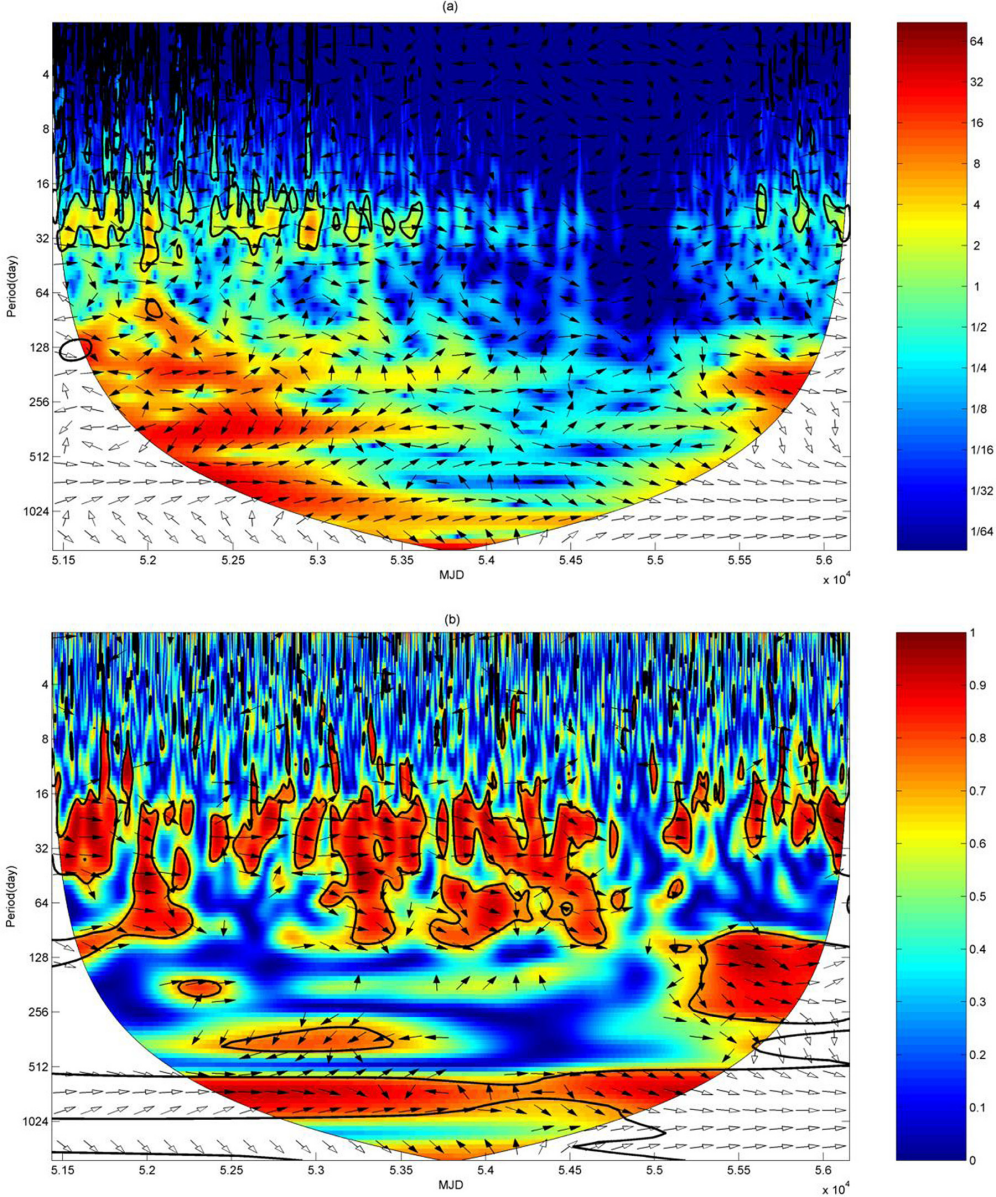
Cross wavelet power and the wavelet coherence spectrum of SSN and TEC from MJD 51179 to 56403. in which the right arrow represents the same phase, the down arrow represents the phase leading 90 degrees, the left arrow represents the anti-phase, and the up arrow represents the phase leading 270 degrees. (a) is the cross wavelet power spectrum. (b) is the wavelet coherence spectrum.

As shown in Fig 13a, the high energy region of the cross wavelet power spectrum emerges on the scale of 16–32 days from 1999 to 2004 and the cycle passes the significance testing with 95% confidence. The high energy region appears on the scales of 128–256 days and 256–512 days from 2000 to 2004. The high power region is present on the scale of 512–1024 days from 2001 to 2004 but the cycle does not pass the significance testing. So the resonant period of SSN and TEC is 27 days, also shown in Figs 5 and 8, which indicates the effect of SSN on TEC with the main cycle of about 27 days.

As shown in Fig 13b, the high energy region of wavelet coherence spectrum on a scale of 16–32 days exists during all the years studied except years 2008 to 2009, which indicates that the sunspot has a positive relationship with TEC on the scale. Time lag correlation of both time series can be obtained through the calculation of the phase difference. The phase difference of two time series is 30°±12.51° on the scale of 16–32 days, in which 30° means that the phase difference is one-twelfth of the period, and ±12.51° is the convolution error. Therefore, we find that the variation of sunspot is ahead of TEC variation for one-twelfth of the period, or about two days. The high energy regions only exist in local time on the scales of 64–128 days, 128–256 days, 256–512 days, and 512–1024 days, which means that the sunspot influences TEC just in local time on these scales [45].

#### Wavelet coherence spectrum analysis on typical times

**Fig 14:**
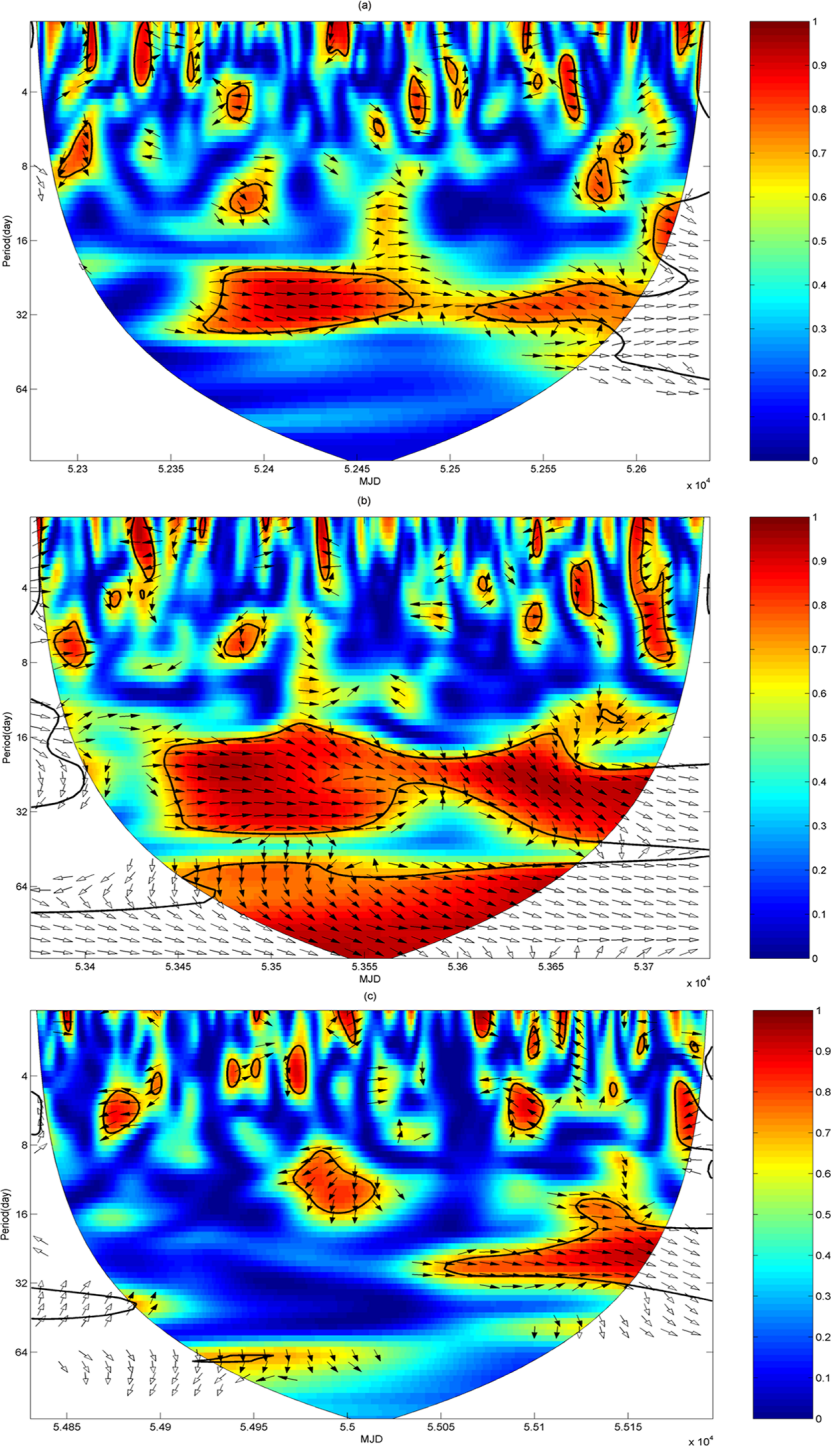
Wavelet coherence spectrum between SSN and TEC in the typical time span. (a) is the wavelet coherence spectrum from MJD 52275 to 52639. (b) is the wavelet coherence spectrum from MJD 53371 to 53735. (c) is the wavelet coherence spectrum from MJD 54832 to 55196.

In order to analyze the differences of the wavelet coherence spectrum in each period, three typical times are selected from MJD 51179 to 56403, that is, MJD 52275–52639 in 2002, MJD 53371–53735 in 2005, and MJD 54832–55196 in 2009. The wavelet coherence spectrums of these three typical times are shown according to the intensity of solar activity in Fig 14.

As shown in Fig 14, most of the high energy regions of the wavelet coherence spectrum exist on the scale of 16–32 days and pass the significance testing, which means that the 27-day period of the sun’s rotation influences the periodic variation of TEC. The solar activity, including the sunspot with the strong magnetic field, the solar flare, and the bright eruption, can generate more ultraviolet rays and X-rays, which are the governing source to become electrons. The solar activity can also generate the high-speed solar wind to influence the Earth’s magnetosphere to result in molecular dissociation [38]. The sunspots are transmitted to the Earth every 27 days because of the sun’s rotation, so the radiation has a cycle of 27 days to make the TEC variation with the period of 27 days.

Most of the high energy regions during the period of MJD 52275–52639 mainly exist in two time spans as MJD 52375–52475 (April–June) and MJD 52530–52600 (September–November). Most of the high energy regions during the period of MJD 53371–53735 exist in the time span of MJD 53450–53700 (March–September), and most of the high energy regions during the period of MJD 54832–55196 exist in the time span of MJD 55060–55150 (September–November). The months from March to November correspond to spring, summer and autumn. The solar activity is severe and the correlation of SSN and TEC is higher during the period, which means that the dependency of TEC on sunspots is seasonal. At the same time, the solar activity in these three periods changes gradually from strong to weak in turn. But the high energy region of wavelet coherence spectrum in the period of 53371–53735 is largest, next is the period of 52275–52639, and the least is the period of 54832–55196. This indicates that the correlation between SSN and TEC is nonlinear. The correlation between SSN and TEC is best when the solar activity is stable.

## Conclusions

The global TEC data estimated from IGS GPS data from 1999 to 2013 are used to analyze the spatial distribution of global TEC. The periodic variations of TEC are analyzed with the wavelet analysis and the least squares spectral analysis. Furthermore, the correlation between TEC and SSN based on the cross wavelet analysis and the wavelet coherence spectrum analysis, as well as the trend of global TEC during this period of 15 years are also studied in this paper.

TECs diminish gradually from the low-latitude region to high latitudes in the spatial distribution. TEC in the mid-high latitude region of the northern hemisphere is less than that in the same regions of the southern hemisphere. The maximum amount of TEC in the world appears in the equatorial crest latitude, the minimum amount of TEC appears in Greenland and its surrounding area. Moreover, TEC over the equatorial region has the two-peak anomaly and is consistent with the geomagnetic equator.

The TEC annual variation from 1999 to 2013 shows a complete primary period similar to the Schwabe cycle. The time interval between the peak year and the trough year for TEC is about 7 years. TEC in spring and autumn is significantly higher than in other seasons. In addition, the winter anomaly is observed in the equatorial region in the northern hemisphere, which is consistent with other research, but the winter anomaly also appears in high latitudes in the southern hemisphere, such as in Antarctica.

TEC has significant 1-day, 26.5-day, semi-annual, and annual cycles. The 1-day, 26.5-day and semi-annual cycles are significant in all time spans, but the annual period is significant in the local time. The fitting results show that the strength of dependency between SSN and TEC has a negative relation with latitude. The dependency is strongest in low latitudes, and TECs in the southern hemisphere are more sensitive to solar activity than that at mid-high latitudes in the northern hemisphere. However, the solar activity sensitivity of TEC is stronger at low latitudes in the northern hemisphere than in the southern hemisphere. Besides, the important resonant period between SSN and TEC is about 27 days, which is seasonal, based on the wavelet coherence power. In general, the sunspot takes place ahead of TEC change by about two days.

TECs over most regions present a decreasing trend during these 15 years based on the least squares spectral analysis, but the TEC over Greenland represents an increasing trend with 0.3 TECu annually, as shown in Fig 9. Furthermore, The effect of solar activity on TEC is analyzed based on the wavelet coherent power, and the result is nonlinear in 2002, 2005, and 2009, as shown in Fig 14. The correlation is maximum when the solar activity is steady.

## Supporting Information

S1 Table Statistics of residuals in the least squares sense. (DOC)
