# Supplementary material for: Temporal-Spatial Variation of Global GPS-Derived Total Electron Content, 1999–2013
Source: PLoS One. 2015 Jul 20;10(7):e0133378. doi: 10.1371/journal.pone.0133378 (PMC4508092; doi:10.1371/journal.pone.0133378)
Supplement: S1 Table — (DOC) [file pone.0133378.s001.doc]

**S1 Table**

| **Statistics** | **Value(0.1TECU)** |
| --- | --- |
| **Max** | 132.31 |
| **Min** | -216.35 |
| **Mean** | -0.0003 |
| **RMS** | 44.49 |
| **Correlation coefficient** | 0.89 |
